# Imaging shear stress distribution and evaluating the stress concentration factor of the human eye

**DOI:** 10.1038/srep08899

**Published:** 2015-03-10

**Authors:** S. Joseph Antony

**Affiliations:** 1 Institute of Particle Science and Engineering, School of Chemical and Process Engineering, University of Leeds, Leeds LS2 9JT, United Kingdom

## Abstract

Healthy eyes are vital for a better quality of human life. Historically, for man-made materials, scientists and engineers use stress concentration factors to characterise the effects of structural non-homogeneities on their mechanical strength. However, such information is scarce for the human eye. Here we present the shear stress distribution profiles of a healthy human cornea surface in vivo using photo-stress analysis tomography, which is a non-intrusive and non-X-ray based method. The corneal birefringent retardation measured here is comparable to that of previous studies. Using this, we derive eye stress concentration factors and the directional alignment of major principal stress on the surface of the cornea. Similar to thermometers being used for monitoring the general health in humans, this report provides a foundation to characterise the shear stress carrying capacity of the cornea, and a potential bench mark for validating theoretical modelling of stresses in the human eye in future.

Stress concentration^1^ occurs in materials when they possess structural discontinuity or anisotropy; either naturally or induced, under different loading environments. In engineering design practices, this is commonly characterised using stress concentration factors (SCFs) depending on the type of loading (normal and/or shear SCFs). SCFs are a key safety indicator in the design of materials and ensure the stability of structures under different functional environments^1^. For example, window panels of the aircrafts are designed to avoid sharp edges to maintain the stress concentration factors within allowable limits. This has helped to reduce the number of air accidents due to structural failures quite dramatically^2^. Unlike in the case of man-made materials, information on the shear stress distribution characteristics and estimates of shear stress concentration factors of the human eye is scarcely found in the literature.

Here we provide in-vivo distribution of shear stress and stress concentration characteristics on the surface of a healthy human eye. Tests are conducted on a typical left eye of a healthy, normal white male subject in the 40–45 years age group, with no other known defects. The study is based on measuring birefringence in the cornea^3^ (Fig. 1) using photo stress analysis tomography (PSAT), a method well known in engineering^4^ and medical applications^5^ for visualising stresses in birefringent materials. Recent developments in the digital imaging and processing of birefringent data provide the opportunity to visualise shear stress distribution whole-field in the eye. This is advantageous as the birefringent measures can be quantified for any point of interest on the surface of cornea in relation to its optical axes. Existing methods of measuring stress in the eye for example, measuring an intra-ocular pressure (IOP) only provides an average pressure (i.e., force required to applanate target area of the cornea)^6^. Furthermore, IOP provides the fluid pressure inside the eye, and hence is hydrostatic in nature (about 22 mm of Hg, and a slight variation of this is possible in the normal eye^6^ at different times of the day or seasons). However, under normal working conditions, biological materials such as the human cornea tissue could experience non-hydrostatic shear stress distribution and this aspect is not yet fully understood. Hence the focus of this current work is based on measuring the maximum shear stress (anisotropic) distribution on the surface of the cornea in vivo. Failure of the materials is known to show more dependence on the anisotropic state of stress fields than on the hydrostatic state of stress^7^. However, hydrostatic loading on an anisotropic structural body, such as an eye cornea, could still experience anisotropic stress distribution. It is important to assess it to improve our fundamental understanding of the functioning and health of the human eye.

Birefringence in materials refers to having different indices of refraction at different crystallographic orientations at the point of interest under the mechanical stress^4^,^8^. The cornea is the front window of the human eye^8^,^9^,^10^ (Figure 1). Ocular media of the human eye^8^,^10^ (i.e., the transparent substances of the eye including the cornea, the aqueous humour, the crystalline lens and the vitreous humour) is naturally birefringent^11^,^12^,^13^. Previous studies on birefringent measurements in the human eye have focused mainly on the medical treatment perspectives for example, to characterise retinal structures^14^, their age effects^14^ and in vitro analysis of keratoconus^15^. A number of studies based on X-ray diffraction have provided an insight into the structural architecture of the human eye^12^,^13^,^14^,^15^,^16^. They report anisotropy in the orientation of collagen fibrils (diamond-like structure)^16^ which provides mechanical stability of the cornea^12^,^15^. Computational^17^,^18^ and analytical^19^ studies are reported to estimate the biomechanical properties of eye tissues, but their model parameters are still being disputed^20^. New experimental results on shear stress profiles of the eye could help to fine-tune the bio mechanical simulation parameters of cornea.

Here we present an engineering approach to understand the shear stress distribution characteristics of the human eye using reflection type PSAT^4^,^21^. The working principle of PSAT is outlined in Fig. 2 (also refer to the method section). In brief, an achromatic, fully circularised polarised light falls on the surface of the cornea. The surface of the cornea has a curvature, although relatively small when compared with other parts of the eye (Fig. 1). Hence using a circularly polarised light to ‘sweep’ the surface of cornea helps to minimise the error in the retardation measurements as it is orientation independent and allows for making birefringent measurement at all orientations simultaneously^22^ (more corrections would have been required if the falling light was plane-polarised only). The tomography is designed efficiently to minimise any such errors for practical purposes^21^. Previous studies have also reported that the human cornea exhibits circular birefringence^23^,^24^. We show later that the current birefringent measurements on the cornea are comparable with using other methods reported in the previous literature, for example an optical coherence tomography^12^. The out-coming light is elliptically polarised and carries the signature of birefringent retardation of the cornea. The elliptically polarised light is examined using an analyser for a number of angular positions of the optical axis of analyser (α) with respect to the major axis of the ellipse (Fig. 2). The outputs are used to build the birefringent map of the cornea and used subsequently in the stress analysis (see the method section).

## Results and Discussion

The components (τ_1_ and τ_2_) of the maximum shear stress (τ_max_)^7^,^21^ are illustrated in Fig. 3. These, together with the direction of the major principal stress at any point of interest on the cornea, provide the temporal and spatial distribution of shear-bearing regions of the cornea. Figure 4 presents the in vivo sensing of shear stress acting on the horizontal and vertical planes of the cornea (τ_1_). The values of retardation reported here (and the mean value 45 nm) using light wavelength 650 nm are comparable to and within the range of previous studies on birefringence measurements of the human eye, using other methods in a different context: 0–95 nm using a wavelength of 585 nm (mean approx. 50 nm) and 30–69 nm at a wavelength of 828 nm using polarisation sensitive optical coherence tomography (PS-OCT)^12^. This supports the adequacy of using PSAT in the current research reported here. Interestingly, the shear stress profile quantitatively shown in Fig. 4 correlates well with previous studies reported on the diamond-like structural anisotropy of normal cornea using X-ray based studies^16^ and close to the diamond-like manual (qualitative) traces of fibril structures^22^ in the eye. Previous studies have shown that the diamond-like structure contributes to the structural stability of the eye^12^,^15^. Hence the birefringent measurements made here are consistent with previous literature but, more importantly, the current alternative approach is non-X-ray based and possesses no additional health risks to the eyes. From the stress analysis, it is evident that structural architecture of the cornea is active in sustaining the shear, and correlates to the distribution of shear along the horizontal and vertical planes (shear stress architecture). The measurement of shear stress provided here in Fig. 4 can also be calibrated to the Pa unit for which further mechanical properties of the cornea are required. For example, based on previous literature, the following parameters are considered: Young's modulus and Poison's ratio of cornea are 1 MPa and 0.49 respectively^17^,^18^. Average cornea thickness 520 μm^18^ and maximum shear stress at the centre of the cornea ~62 μPa^25^. Based on these, a multiplication factor of 3.175 is to be applied to scale the retardation to the μPa unit. However, this conversion would not affect the determination of the eye stress concentration factors reported here (see method section).

The stress-structural anisotropy correlation, as observed above, is also supported by probing the directional alignment of the major principal stress on the surface of the cornea. This is presented in Fig. 5. A detailed analysis was performed on the individual distributions of τ_max_ and τ_2_, and their profiles are also presented here. We observed that the profile of the maximum shear stress distribution displays diamond-like architecture. The shear stress distribution profile at 45° plane to the horizontal and vertical axes (τ_2_) displays a comparable level of activity but tends to possess relatively less anisotropy when compared with the τ_1_ distribution. Overall, it is evident that the diamond-like architecture of shear stress profiles along the key planes viz., horizontal and vertical planes and the plane carrying maximum shear stress are similar to the structural architecture of the cornea reported earlier^16^,^22^. They enlighten the flexibility of the corneal tissues possess to enable the structural movements of the eye, and an in-built ability to distribute shear at orthogonal directions.

Furthermore, similar to the evaluating stress concentration factors as a design parameter in engineering materials and structures^4^,^7^, a strength analysis is performed by examining the signatures of τ_max_ at different sections of the cornea to quantify eye shear stress concentration factor K_e_ defined as follows:

Maximum shear stress τ_max_ = K_e_ τ_0_

K_e_ = eye shear stress concentration factor (eSSCF)

τ_0_ = Nominal value of maximum shear stress

Figure 6 presents the variation of eSSCF in the cornea along the superior-inferior axis (section 1–2), nasal-temporal axis (section 3–4) and the 45° orthogonal axes (sections 5–6 and 7–8). The maximum value of eSSCF (w.r.t. point1) along these axes is found to be 2.60, 2.50, 2.76 and 2.38 respectively. By and large, the eSSCF profiles tend to present symmetry along the superior-inferior axis from the middle of the cornea, but to a relatively less extent in the case of other axes. For comparison purposes, the variation of eSCCF along the sections is also provided by considering the nominal shear stress as equal to the average value of τ_max_ corresponding to the chosen sections. The difference in the plots of eSCCF between the two cases of accounting for the nominal shear stress is relatively strong along the 1–2 and 5–6 sections of the cornea. However, this difference tends to diminish in the case of 3–4 and 7–8 sections. This suggests that the corneal tissue arrangement that bears the shear is strongly anisotropic along the superior-inferior vertical axis and the diagonal axis on the nasal side. Interestingly, the values of eSSCF are comparable to some cases of shear stress concentration factor of engineering structures, for example, sheared shallow spherical shells with small holes^26^ and geometrical discontinuities in shafts^27^. The variations of eSSCFs further confirm the strong mechanical anisotropy of the human cornea in transmitting shear stress in the eye. It should be pointed out that the current measurements of shear stress concentration factors of the cornea are based on effective (resultant) shear stresses- i.e., combined effects of any inherent stresses present within the molecular(/structural) architecture of the cornea during its formation and the induced stresses on it. It may be worth quantifying these components separately for future studies.

### Conclusions and scope for the future

To summarise, the cornea is a structurally complex element which shears in its plane^28^. However, the shear stresses distribution characteristics of the human cornea are not yet well understood, and are addressed in this present work. The employed method of measuring shear stress distribution and eye stress concentration factors is simple and non-X ray based, therefore friendlier to eye tissues. Many of the previous biological studies measured retardation at selected points (or sections) of the cornea. However the current approach used in this work resulted whole-field measurements. The current measurement of corneal retardation is within the range of previously reported results in the literature. Furthermore, the present measurements are made in vivo which is advantageous because in vitro experiments in the eye might not truly reflect the stress gradients of the structural elements in the cornea^8^. The shear stress distribution at both the horizontal and vertical planes, and also at the plane carrying maximum shear stress of the cornea is shown to have a good level of correlation with the diamond-like architecture of corneal tissues^16^ (Figs. 4, 5). The results show that the cornea is effective in distributing shear in the horizontal and vertical planes, as well as at 45° to the principal stress direction (where maximum shear stress acts). Overall, the present stress analysis demonstrates the effectiveness of the structural architecture of the cornea (diamond-like at^16^ or close to^22^ 45° angles of superior-inferior and nasal-temporal directions) in distributing shear loading. This is a naturally favourable stress distribution mechanism for easing the movements of the eye in daily life. The stress concentration factors show non-uniform variation along the different sections of the cornea as studied here, but the profiles tend to be relatively more symmetric along the superior-inferior section, passing through the centre of the cornea. The stress profiles presented here could aid modellers to compare with, and calibrate the material parameters of the cornea in their simulations. This is urgently required, as bio mechanical model parameters of eye elements are still uncertain in existing literature^20^. For example, studies have shown that the measurement of the cornea thickness could depend on the type of equipment used^29^. Measurements of material parameters, such as elastic modulus, could vary within the corneal tissues at molecular and micro scales^30^,^31^. Their effects on the collective ability of the cornea to sustain shear is still not well known. However, the present whole-field and in vivo stress measurements and analysis provide a novel foundation with an engineering approach to characterise a complex biological problem. By employing the methodology used here for mapping out the shear stress distribution profiles at different planes using Mohr's circle, the stress measurements could be linked with new optimisation and inverse calculation methods^32^ to fine tune the material parameters (e.g. elastic modulus) and model architecture in such a way that the simulations could initially reproduce the experimentally measured shear stress distribution characteristics of a healthy cornea. Thereafter, the sensitivity analysis of the material parameters on the shear stress distribution characteristics of other environmental conditions of the eye could be examined using simulations with relatively more confidence.

The present study is not without limitations. More studies are also required to obtain a wider representation of the stress intensity profiles in the human eye. Future collaborative studies with the participation of engineering and medical researchers, could investigate the corneal stress distribution characteristics for a wider representation of subjects, age groups, gender and ethnicity and in other stress-related environments. Such efforts could lead to developing additional post-operative eye health compliance criteria by comparing the stress concentration factors at different sections of the structural elements of the normal and abnormal cornea (in addition to corneal topography) in vivo. Abnormal stress raisers, using the stress concentration profiles of the cornea, could be identified in treating patients where stress effects could change the birefringence in the cornea, for example, in keratoconus patients^15^. The present study has not considered any variations in the IOP on the shear stress distribution characteristics and the shear stress concentration factors of the eye. Minor variations in the IOP of normal eyes are less likely to change the values of eSSCFs significantly as they are relative measures. However, more detailed studies could be conducted in future taking into account the potential effects of IOP variations on anisotropic stresses during different periods of the day especially where the cornea is abnormal. In addition, development of eSSCFs due to infectious eye irritations, injuries and fatigue due to aging are also required. Fatigue failures usually originate from stress raisers, so removing such defects could improve the failure strength and quality of the imaging ability of the human eye. As the present method does not involve using X-rays, the methodology is particularly suited for making repetitive stress measurements in the cornea for example, measuring stresses at different time intervals without any significant risk in the future. The potential opportunities are extensive.

## Methods

**Figure 2:**
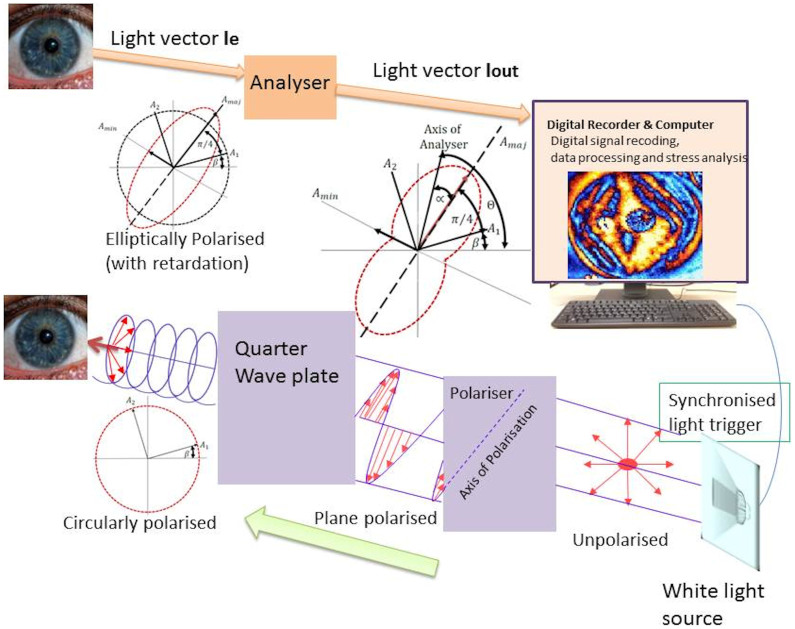
Schematic diagram of the working principle of PSAT. See the method section for more details (Thanks: J. Drewes for the eye image insert).

**Figure 3:**
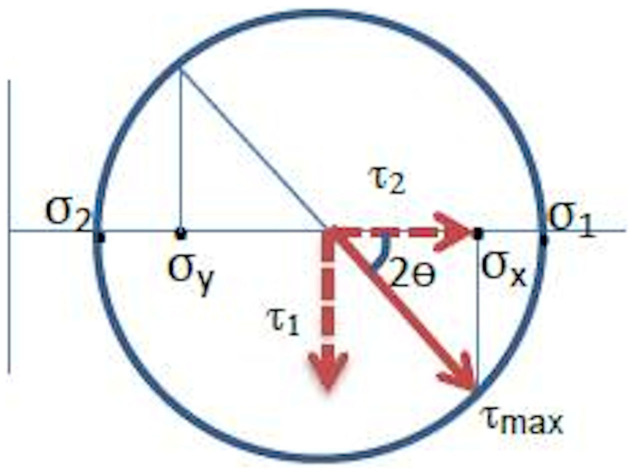
Components of maximum shear stress τ_max_ marked on the Mohr's circle^7^,^21^ representing shear stress state at a point of interest on the cornea. τ_1_ represents shear stress acting in horizontal and vertical planes whereas τ_2_ represents shear stress acting at 45° to the horizontal and vertical planes. τ_max_ is the maximum shear stress acting at 45° to the principal stress direction. σ_1_ and σ_2_ are the major and minor principal stresses respectively.

**Figure 4:**
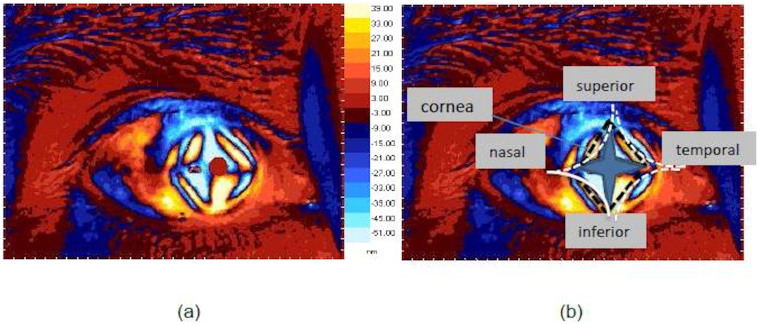
(a) Distribution of shear stress τ_1_ (in the unit of retardation^4^) and (b) same picture as in (a), but with trend lines of the direction of collagen fibrils^16^ superimposed. Fibrils are the basic structural elements of human eye. In general, about 60% of fibrils are oriented along the 45° orthogonal directions (shown as diamond-like structure^16^ in dotted lines) and the remaining fibrils are oriented in between these orthogonal sectors and assist in maintaining the corneal shape^15^.

**Figure 5:**
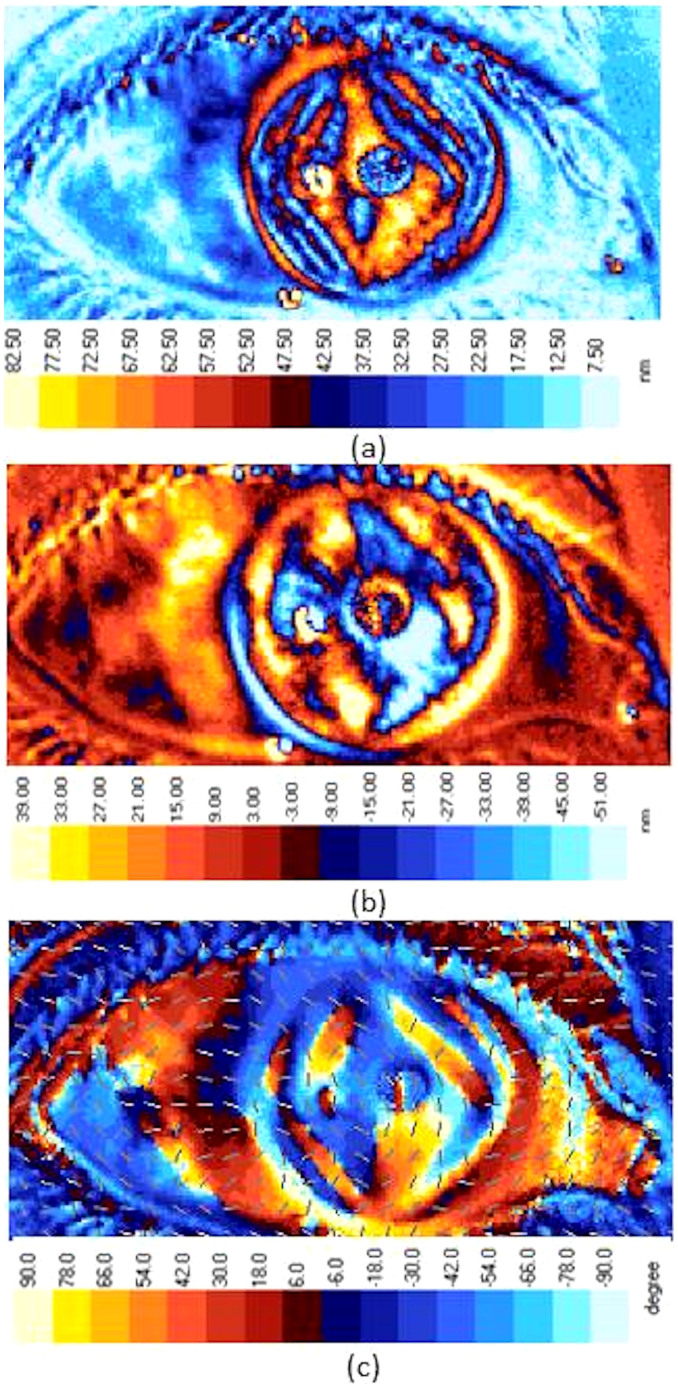
Distribution of shear stress in the cornea: (a) τ_max_ and (b) τ_2_. The direction of the major principal stress is presented in (c) using the guide arrows as well as colour coded. They present a diamond-like structure as presented in Fig. 4, suggesting the active role played by the structural architecture of the cornea^16^ in bearing shear stress.

**Figure 6:**
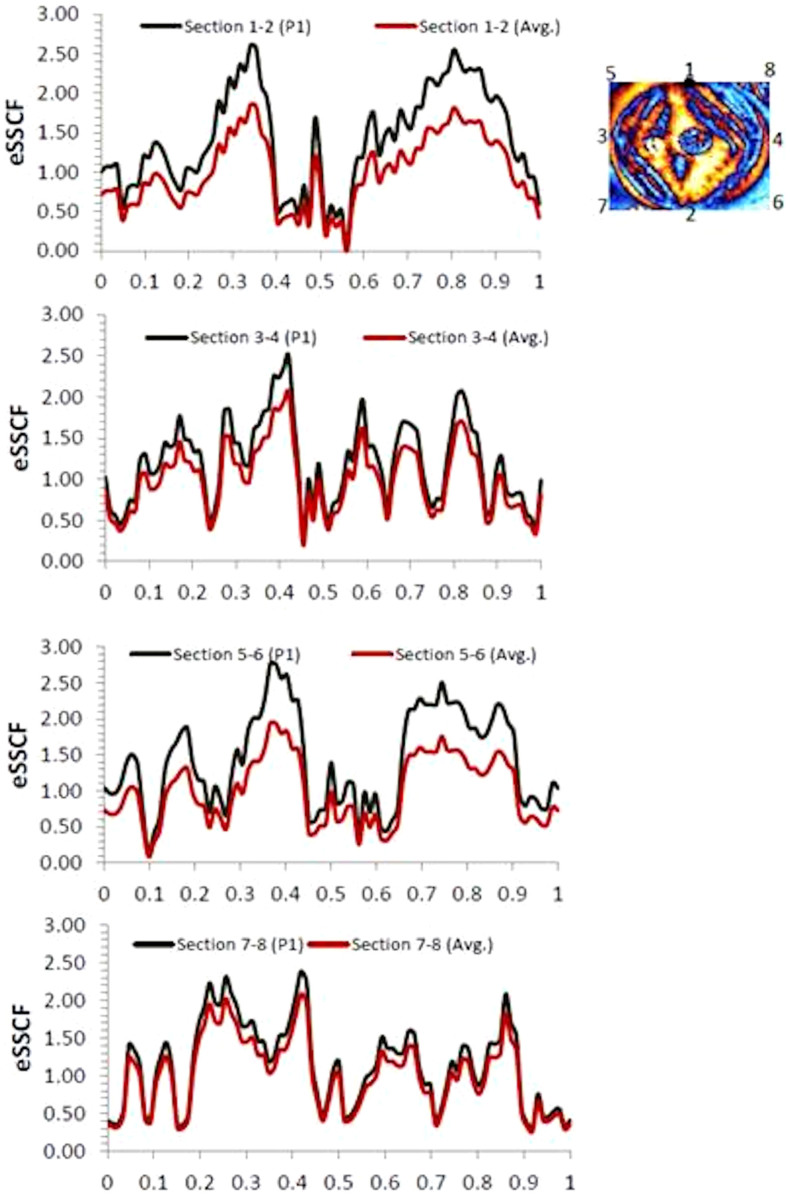
Variation of the shear stress concentration factor (eSSCF) of the cornea. Horizontal axes show the distance normalised to the length of the cornea along the sections. Each plot has two profiles and they differ in the way nominal stresses are calculated. In the first case, it is taken as the value of τ_max_ at point 1(P1) at the beginning of the cornea at the superior side of axis 1–2, and in the second case average value of τ_max_ of the chosen section are considered. Measurements are made at 85 points along each section. The standard deviation value for sections 1–2, 3–4, 5–6 and 7–8 w.r.t. P1 are 0.65, 0.50, 0.59, and 0.56 respectively. In the cases of this w.r.t. the average value is 0.65, 0.41, 0.47 and 0.49 respectively. This outcome helps to understand the anisotropy in the shear stress profiles of the cornea.

The working principle of PSAT, which is a grey field polariscope^21^ (Fig. 2), is based on photoelastic birefringent properties of materials^4^,^8^,^28^. PSAT is a hybrid of a plane-polariscope and a circular polariscope^4^,^21^. It illuminates the target sample (cornea) with circularly polarized light but analyses it with a linear polarizer (Fig. 2). A comprehensive mathematical treatment of its optics can be found elsewhere^21^ but the working principle is briefly outlined here. Initially, a white light source is triggered (light wavelength 650 nm) and passes through the plane polariser. The out-coming light of the polariser is a plane-polarised light with its optical axis aligned along the axis of polarisation. When this passes through a quarter-wave plate with its axes oriented at 45°, with respect to the axis of the polariser, the circularly polarised light emerges out (*A1* and *A2* are the corresponding light components, parallel to and perpendicular to the fast axis respectively). This falls on the cornea. The reflecting light I_e_ from the cornea is elliptically polarised^21^ and carries information on the retardation experienced by the cornea along their fast and slow optical axes. Δ is the phase lag with respect to the fast axis. As in the case of previous studies^8^ on corneal birefringent measurements, individual contribution of other ocular media to retardation is ignored. *A_maj_* and *A_min_* are the light components along the major and minor axes of the ellipse respectively. The major axis of the ellipse is shifted by *π*/4 for odd fringes and −*π*/4 for even fringes^21^. The out-coming polarised light intensity I_out_ is analysed using an analyser (which is optically identical to a polariser), for different optical orientation of the analyser (α) and the signals are digitally recorded. From this captured image of the cornea (comprising birefringent fringes of different orders^4^), the retardation can be resolved along the major and minor principal stress axis at any point of interest on the cornea. This optical information can be related to stress information using the stress-optic law^4^,^33^, which has been used for more than a century in birefringent stress measurements: σ_1_ − σ_2_ = nF_σ_ in which σ_1_ and σ_2_ are the major and minor principal stresses respectively, n is the fringe order (at the point of interest as a result of Δ) and F_σ_ is the stress fringe constant determined by calibration either numerically (for example as described in Fig. 4), or experimentally. However, representing stresses in terms of the retardation unit does not require this calibration. Also determining the direction of principal stress components and the shear stress intensity factor (which is a relative measure) does not require this calibration. Furthermore, the stresses can be expressed using the well-known Mohr's circle representation^4^,^7^. Using this shear stress in the vertical and horizontal planes, together with the maximum shear stress and the direction of principal stresses, can be represented easily^4^,^7^,^21^. The tomography set up is a powerful tool to measure sub fringe measurements accurately (less than 1 nm). The integrated experimental setup with a digital camera and computer allows completion of the retardation measurements in the subject at an adequate speed (about 20 revolutions of analyser positions within a minute) and with high precision lenses so that even a fraction of retardation can be recorded.

## Figures and Tables

**Figure 1:**
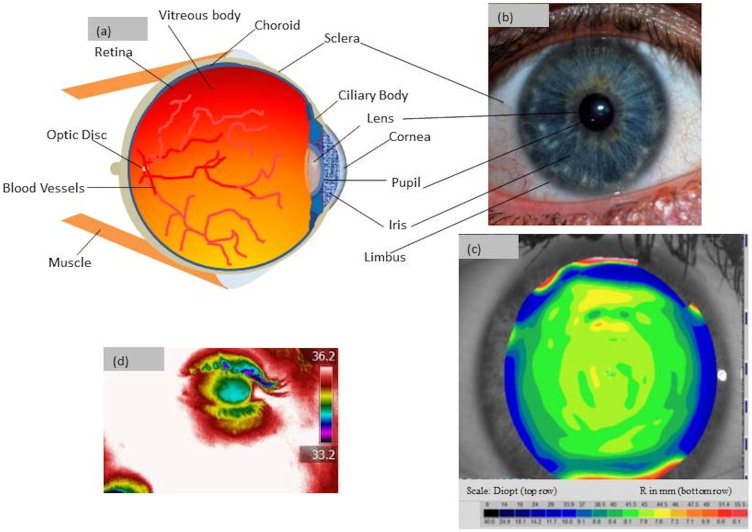
Generic features of a healthy eye: (a) Schematic diagram of eye anatomy (b) visual image (Thanks: J. Drewes) (c) A typical curvature map of a human cornea (anterior), in which R is the radius of curvature (about 7.9 mm in the plane normal to lens^8^,^9^). Diopt is related to the R measurement which is a unit measure of refractive power of the cornea^9^ and (d) Temperature could vary slightly in the eye with cornea temperature lower than that of sclera region, measured with an Infra-Red camera in °C.
